# Do therapists with fewer years of clinical experience encounter more accidents? The relationship between number of years of clinical experience and number of accidents in a year

**DOI:** 10.20407/fmj.2019-015

**Published:** 2020-07-14

**Authors:** Akiko Maeda, Megumi Suzuki, Toshio Teranishi, Mihoko Ito, Nozomi Hokimoto, Kenta Fujimura, Hirofumi Ota, Eiichi Saitoh

**Affiliations:** 1 Faculty of Rehabilitation, Fujita Health University, School of Health Sciences, Toyoake, Aichi, Japan; 2 Department of Rehabilitation Medicine I, Fujita Health University, School of Medicine, Toyoake, Aichi, Japan

**Keywords:** Accident prevention, Patient safety, Rehabilitation, Therapy

## Abstract

**Objectives:**

This study sought to determine whether therapists experience more accidents annually with increased clinical experience, and whether experiencing an accident in the first year of practice is associated with accidents in the second year of practice.

**Methods:**

We categorized 642 therapists into five groups based on years of clinical experience (first, second, third, fourth, and 5–20 years; n=138, 112, 117, 58, and 217, respectively) and tallied the accidents they reported over an 8-year period. The difference between the five groups in the number of accidents per person per year was subjected to multiple comparisons testing using Kruskal–Wallis tests.

**Results:**

Significant differences were revealed between the first year group and the 5–20 years group (p<0.01), between the second year group and the 5–20 years group (p<0.05), and between the third year group and the 5–20 years group (p<0.05). Specifically, participants in the 5–20 years group encountered fewer accidents than those in the other groups. Therapists who encountered an accident in their first year, compared with those who had not, had significantly more accidents in their second year.

**Conclusions:**

Therapists with 1–3 years of clinical experience are more likely to encounter an accident than therapists with >5 years of clinical experience. We conclude that young therapists who have experienced accidents are prone to future accidents. These findings inform the optimal allocation of educational resources to reduce the number of accidents encountered by therapists.

## Introduction

Falls and other accidents during rehabilitation training can delay rehabilitation and cause fear of moving among patients, reducing their motivation to undergo rehabilitation. Reducing accidents during rehabilitation training is important for helping patients smoothly rejoin the community. Therefore, many studies and programs have examined the types, causes, frequency, and prevention measures regarding falls and other accidents.^1–4^ For example, Fukue examined initiatives for preventing falls in a convalescent rehabilitation ward.^1^ Meanwhile, Ganz reported the prognostic value of risk factors for future falls among older patients.^2^ Recently, Inoue investigated whether falls could be reduced by implementing a fall prevention program for new therapists.^3^

Here we report the results of an investigation of the occurrence of accidents during rehabilitation training at advanced treatment hospitals, which are hospitals with the capacity to provide advanced medical care, develop advanced medical care technology, and hold workshops on advanced medical care. The most frequently occurring accidents during rehabilitation training at advanced treatment hospitals are, in order of occurrence, bleeding, falls, and route-related accidents (i.e., trouble with drip infusions or urinary catheters), and approximately half of these accidents occur in the training room. Bleeding can occur while lying down, sitting, walking, or performing other actions, and 43.9% of falls occur while walking.^5^

Many studies have proposed that the occurrence of accidents is causally related to the number of years of clinical experience or other characteristics of therapists, reporting a greater number of accidents involving medical staff with less clinical experience compared with more experienced staff.^5–8^ However, several of these studies only examined the number of accidents or the number of medical staff members involved in an accident. Thus, the size of the population of therapists who have not encountered accidents remains unclear, and few studies have statistically verified the number of accidents involving young medical personnel by adjusting for the population of therapists who have encountered an accident as well as those who had not. Furthermore, previous studies have not tracked individual therapists over time to determine the influence of encountering or not encountering an accident each year on the number of accidents occurring in the following year.

Therefore, by focusing on physiotherapists, occupational therapists, and speech-language-hearing therapists at advanced treatment hospitals, the purpose of the current study was to determine whether the number of accidents encountered by therapists in a year changes with an increase in the number of years of clinical experience. We also investigated the effects of encountering an accident in the first year of clinical experience on the number of accidents encountered in the following year. Our findings may be useful for informing the optimal allocation of educational resources, and the development of strategies to reduce the number of accidents encountered by therapists.

## Methods

### Participants

Participants were 236 therapists who had worked at an advanced treatment hospital with 1,435 beds for at least 1 year between April 1, 2009 and March 31, 2017. The participants consisted of 119 physiotherapists, 84 occupational therapists, and 33 speech-language-hearing therapists.

In total, 46,380 patients underwent rehabilitation training at the advanced treatment hospital over the 8-year study period. The proportions of patients receiving rehabilitation training in various departments were as follows: 11.9% in orthopedic surgery, 8.8% in cerebral stroke care, 8.6% in emergency care, 7.6% in respiratory medicine, 7.2% in neurology, 6.3% in gastroenterology, 4.9% in cardiovascular medicine, 3.9% in cardiovascular surgery, 2.6% in nephrology, 2.5% in otorhinolaryngology, 2.4% in obstetrics and gynecology, 2.3% in emergency and critical medicine, and 23.9% in other departments.

This study was approved by the Fujita Health University Ethics Review Committee (approval no. HM17-083).

Therapists were divided into five groups based on the number of years of clinical experience: first year, second year, third year, fourth year, and 5–20 years. The five groups were classified as follows: therapists in their first year of clinical experience were categorized as having less than 1 year of experience, those in their second year were classified as having 1 to 2 years of clinical experience, those in their third year were classified as having 2 to 3 years of clinical experience, those in their fourth year were classified as having 3 to 4 years of clinical experience, and those in their 5–20 years were defined as having 4 to less than 20 years of clinical experience. A few therapists who had more than 20 years of clinical experience were excluded from the analysis because the sample size of these therapists was very small, and the data would have strongly reflected the characteristics of specific individuals. Next, the number of accidents for each therapist and the number of years of clinical experience of the therapist when they encountered the accident were obtained for each of the 8 years from reports submitted by the therapists at the time of each accident.

### Comparison of the number of accidents encountered per year per therapist

The Kruskal-Wallis test was used to compare the number of accidents encountered per year per therapist between the five groups. Multiple comparisons were performed using Dunn’s multiple comparisons test.

The statistical analyses were performed with SPSS 24.0 (SPSS Inc.; Chicago, IL, USA), and the level of significance was set at <0.05.

### Relationship between accident encounters in the first year and the number of accidents encountered in the following year

The 101 therapists who were at the hospital for at least the first 2 years of their clinical experience were divided into two groups based on whether they had encountered an accident in their first year. Next, the Mann-Whitney U test was performed to examine the differences between the two groups in the number of accidents encountered per year per therapist in the following year.

These statistical analyses were performed with GraphPad Prism 7.0 (GraphPad Software, San Diego, California, USA), and the significance level was set at <0.05.

## Results

The 236 therapists who participated in this study were divided into five groups based on the number of years of clinical experience (Table 1). In total, there were 578 reports of accidents among the therapists over the 8-year period, including 158 among those in their first year, 128 among those in their second year, 106 among those in their third year, 60 among those in their fourth year, and 126 among those in the 5–20 years group.

### Comparison of the number of accidents encountered per year per therapist

**Figure 1:**
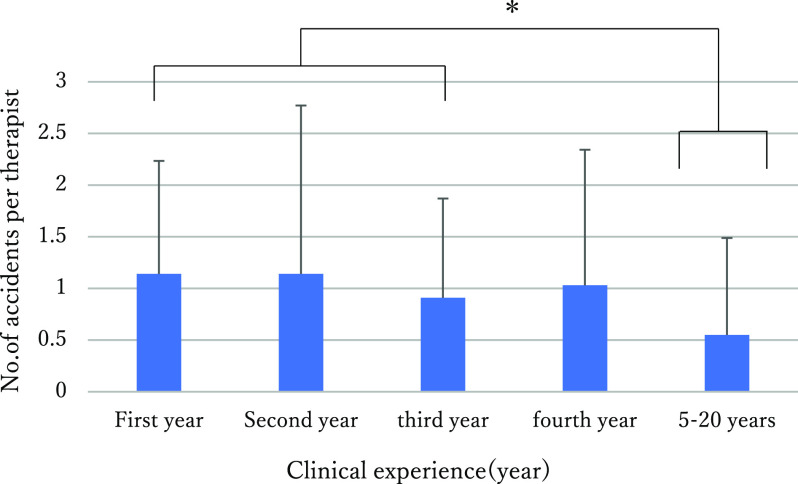
Annual number of accidents per therapist by years of experience. Multiple comparisons between the five groups (Kruskal-Wallis test) revealed significant differences between the first year group and the 5–20 years group (p<.01), between the second year group and the 5–20 years group (p<.05), and between the third year group and the 5–20 years group (p<.05); specifically, therapists in the 5–20 years group encountered fewer accidents than those in the other groups.

The number of accidents encountered per year of clinical experience per therapist was 1.149 for those in their first year, 1.142 for those in their second year, 0.91 for those in their third year, 1.03 for those in their fourth year, and 0.44 for those in the 5–20 years group (Figure 1).

Concerning multiple comparisons between the five groups (Kruskal-Wallis test), significant differences were revealed between the first year group and 5–20 years group (p<0.01), between the second year group and the 5–20 years group (p<0.05), and between the third year group and the 5–20 years group (p<0.05); specifically, therapists in the 5–20 years group encountered fewer accidents than those in the other groups.

### Relationship between accident encounters in the first year and the number of accidents encountered in the following year

Sixty-seven therapists encountered an accident in their first year, and 34 did not. The number of accidents per capita in the following year per therapist in the group who encountered an accident in the first year was 1.07. The number of accidents per capita among therapists who did not encounter an accident in their first year was 0.56 in the following year. A comparison of these two groups using the Mann-Whitney U test revealed that the number of accidents encountered in the second year was significantly higher among therapists who had encountered an accident in their first year compared with those who had not (p<0.05; if an accident occurred in the first year: median 1, if no accident occurred in the first year: median 0; Z value=–1965).

The breakdown of the types of accident among therapists in the first-year group were as follows: bleeding=37.8%, falls=23.0%, route-related accidents=23.0%, other=16.2%. Among therapists in the second-year group, these rates were 27.4%, 35.6%, 30.1%, and 6.8%, respectively. Forty of the 67 therapists who had encountered an accident in their first year encountered an accident the following year; of these, 19 therapists encountered the same type of accident.

At the time of the accident, 58.1% of the 67 therapists who had encountered an accident in the first year of clinical experience were involved in an accident during supervised practice where they did not touch the patient directly, and 35.1% of them were involved in an accident during assisted practice (6.8% of therapists encountered accidents in a different situation). Among therapists in their second year, these rates were 56.2%, 37.0%, and 6.8%, respectively. Comparisons between the two groups using chi-squared tests revealed no significant differences.

## Discussion

A wide range of the number of years of clinical experience (1–20 years) was observed among the therapists. The distribution of clinical experience of therapists was similar to that of the population of certified therapists in Japan, and there were more therapists with fewer years of clinical experience.^9^ This suggests that the composition of the study population was similar to that of therapists in Japan in general. Furthermore, because we analyzed data obtained over an 8-year period, the reliability of the information was high.

In a previous study, Higashi reported the actual state of risk of accidents in occupational therapy and other rehabilitation training settings from 2003 to 2004.^6^ In terms of years of experience, Higashi reported that there were seven accidents involving therapists with less than 1 year of clinical experience, five involving therapists with 1–5 years of clinical experience, and one involving therapists with 6–10 years of clinical experience. This finding suggests that therapists with less clinical experience are more likely to encounter accidents; however, Higashi’s report did not specify whether the study included therapists with more than 10 years of experience.^6^ Thus, Higashi’s findings suggested a tendency for therapists with fewer years of experience to encounter more accidents, in accord with the current results. However, there have been many other reports on accident frequency and years of clinical experience, producing variable findings.^6–8,10^ The method typically used in these previous studies is to count the number of accidents occurring for the number of years of clinical experience based on accident reports; this approach has typically led to results indicating that a higher number of accidents are encountered by therapists with fewer years of clinical experience. However, the number of therapists who have not encountered an accident is unclear in studies using this design; therefore, it is possible that the samples include a greater number of therapists with fewer years of clinical experience, and this group could comprise a larger proportion of the data. As such, counting the number of accidents in the number of years of clinical experience alone cannot confirm that less clinical experience is related to more accidents.

Concerning the results of the present study, although therapists with 5–20 years of experience encountered significantly fewer accidents compared with therapists in their first, second, and third years, no significant difference was found in the number of accidents between therapists in their first, second, and third years. It should be noted that first year therapists are required to complete a probation period. As such, it is possible that they are placed in charge of patients with less severe symptoms or less challenging risk control than second year therapists. Risk control is necessary for patients who are prone to sudden changes, such as blood pressure fluctuations and those who require route management, such as infusions and balloon catheters. In addition, therapists who are likely to encounter accidents regardless of the number of years of clinical experience may be biased and tend to avoid cases that require strict attention to risk management. However, even if such a bias exists, the number of accidents encountered by the first, second, and third year groups was higher than that encountered by the 5–20 years group.

Furthermore, the number of accidents encountered per therapist in second year was significantly higher among those who had encountered an accident in first year compared with those who had not. In a report on changes in personal characteristics and the tendency of medical errors owing to the number of years of nursing work, Sakai et al.^11^ reported that nurses with less experience have more repeat accidents and higher work stress.

Matsumura^12^ reported the involvement of human error in the occurrence of accidents during rehabilitation. Human errors can be caused by problems with the workplace environment and systems (e.g., lack of medical equipment, procedures, manuals). In addition, although human error tends to be treated as an individual problem, it is important to minimize education and system errors, and to take systematic measures to prevent accidents from occurring in spite of human error. In the current study, therapists who encountered an accident in the first year tended to be more likely to encounter an accident in the following year. The institution examined in the present study was an advanced treatment hospital with many patients in the acute stage of treatment beginning rehabilitation soon after the onset of symptoms when their general condition had not yet stabilized. As symptoms may worsen during rehabilitation, therapists must provide rehabilitation training in a tense environment; thus, therapists often experience substantial tension and fatigue. Therefore, the working environment and the working system of the therapist, the responses of the supervisor and risk manager after the accident, and communication in the workplace are strongly related to the mental state of therapists.

The results of the present study suggest that therapists with less clinical experience tend to encounter accidents more frequently. Therapists with less clinical experience lack knowledge about risk management, and it may be difficult to perform rehabilitation after predicting danger. Moreover, it is extremely difficult to instantly make appropriate decisions when a patient’s condition exhibits sudden changes. In 1999, the United States Institute of Medicine issued a report titled “To err is human: building a safer health system,” expressing the importance of building systems that do not punish individuals for mistakes but instead prevent mistakes from happening in the first place. Currently, many hospitals conduct programs to prevent the recurrence of medical accidents using a systematic check system.^13^ At the target facility of the current study, any therapist who encountered an accident was required to submit an accident report, analyze the cause of the accident with a risk manager, and consider countermeasures to prevent recurrence.

Accidents encountered by therapists with less clinical experience accounted for 80% of falls, bleeding, and route-related accidents. In many cases of falling, when a patient loses balance, the therapist cannot provide adequate support from their location at that moment. Cases of bleeding are typically caused by excessive movement, bleeding from a wound, bleeding from a limb hitting a wall or medical instrument, or walking barefoot. Furthermore, accidents involving routines included cases in which a patient felt uncomfortable withdrawing an intravenous drip, when a patient pulled a drip during an operation, or when a caregiver behaved carelessly.

In addition, many accidents occur under supervision when the patient is not directly touched during therapy. If the risk of falling is predicted, the therapist will need to monitor the patient from a position in which they can respond immediately; it is difficult to avoid danger if the patient and therapist are not properly positioned. Thus, it is necessary to strengthen medical safety education for young therapists, such as setting up environments to minimize danger, understanding the positional relationship between therapists and patients, and promoting knowledge necessary to respond appropriately in dangerous situations.

One limitation of the present study is that accidents during rehabilitation practice may be caused not only by patients but also by therapists. Thus, the relationships between the accident and the therapist’s personality, mental state, work environment, and organizational system should be investigated. We plan to perform further analyses concerning these variables to inform specific medical safety training to therapists in their first year of training and determine whether such training can reduce the number of accidents in therapists’ second year at the hospital.

## Figures and Tables

**Table1:** Number of therapists by years of clinical experience

	First year	Second year	Third year	Fourth year	5–20 years
FY 2009	7	14	12	4	20
FY 2010	10	9	13	6	22
FY 2011	9	8	8	11	21
FY 2012	15	9	16	2	28
FY 2013	21	14	17	5	26
FY 2014	24	20	12	10	27
FY 2015	19	19	19	8	35
FY 2016	33	19	20	12	38
Total	138	112	117	58	217

FY: Fiscal year
